# Supplementary material for: CPK1 activates CNGCs through phosphorylation for Ca2+ signaling to promote root hair growth in Arabidopsis
Source: Nat Commun. 2025 Jan 15;16:676. doi: 10.1038/s41467-025-56008-4 (PMC11733299; doi:10.1038/s41467-025-56008-4)
Supplement: Supplementary file 1 — Supplementary information. [file 41467_2025_56008_MOESM1_ESM.pdf]

Supplementary information

CPK1 activates CNGCs through phosphorylation for Ca^{2+} signaling to promote root hair growth in Arabidopsis

Meijun Zhu[1, 2], Bo-Ya Du[1, 2], Yan-Qiu Tan[1], Yang Yang[1, 2], Yang Zhang[1, 2], and Yong-Fei Wang[1, 2, *]

[1] National Key Laboratory of Plant Molecular Genetics, CAS Center for Excellence in Molecular Plant Sciences, Chinese Academy of Sciences, Shanghai 200032, China

[2] University of Chinese Academy of Sciences, Shanghai 200032, China

[*] Author for correspondence: Yong-Fei Wang (yfw@cemps.ac.cn)

ORCID ID: 0000-0003-2378-0567 (M.Z.); 0000-0002-7593-6335 (B.-Y.D.); 0000-0002-1750-3558 (Y.-Q.T.); 0000-0001-8516-4036 (Y.Y.); 0009-0006-5431-6125 (Y.Z.); and 0000-0003-3139-7701 (Y.-F.W.)

This Supplementary file contains Supplementary Figures 1-10 and Supplementary Table 1.

Supplementary Figure 1

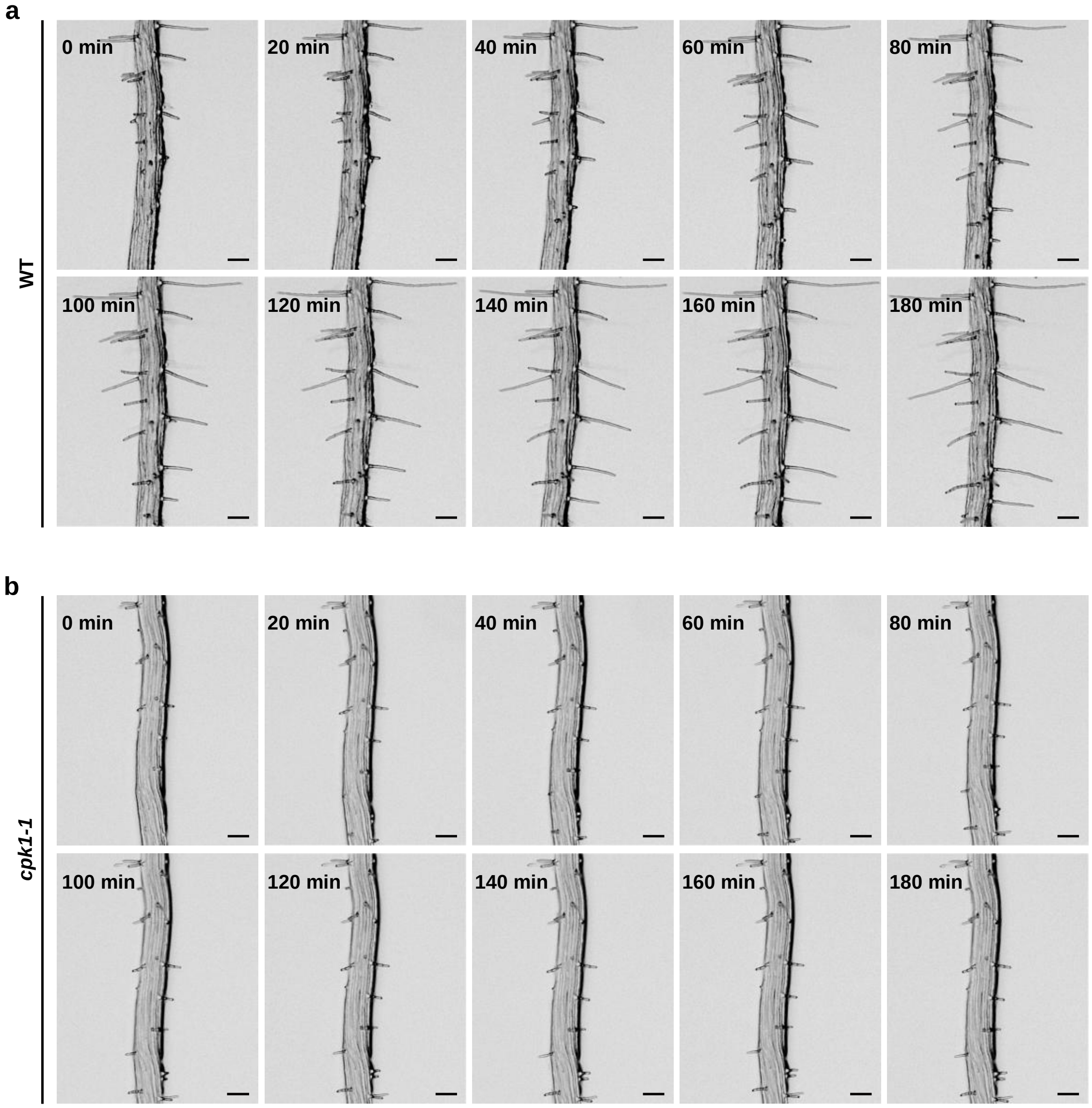

Supplementary Fig. 1 │ Time-lapse images of growing RH in Arabidopsis seedlings. For the time-lapse analysis of RH growth, a set of optical sectioning images were captured under a stereo microscope at room temperature (25 ± 1 °C), and were then merged into a single 2-D photo, each minute. The RHs growing in diverse directions around the primary roots and in the different optical layers can be seen clearly with the same spatial resolution in the merged 2-D photos. The merged 2-D photos were used to monitor the RH growth. **a-b**, Typical merged 2-D photos of the wild

type (**a**) and the mutant *cpk1-1* (**b**). 5 seedlings were analyzed each Arabidopsis line. Scale bars, 0.1 mm.

Supplementary Figure 2

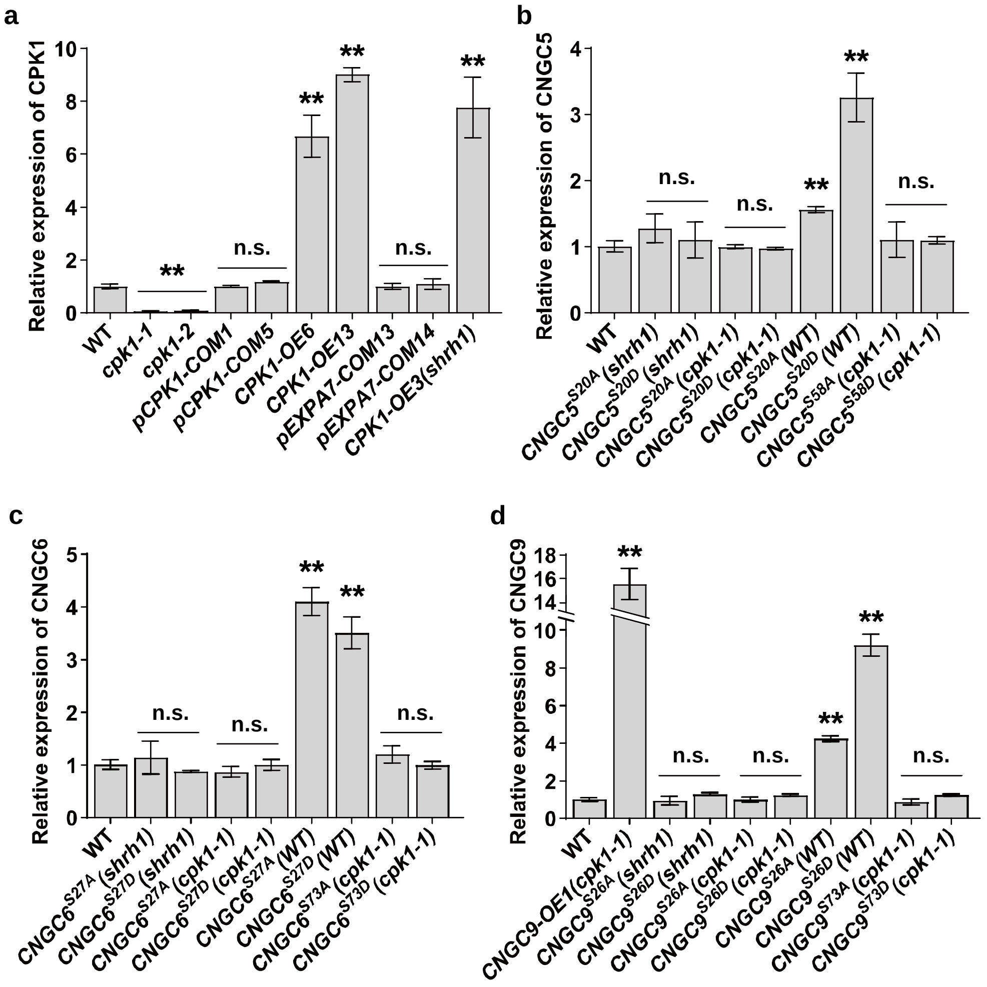

Supplementary Fig. 2 │ RT-qPCR data show the expression levels of CPK1 and CNGC5/6/9 in Arabidopsis lines. Total RNA was extracted from 7-day-old seedlings grown on ½ MS medium in plates to make cDNA, and then RT-qPCR analysis was performed. Three independent biological replicates were used for each Arabidopsis line. The letters n.s. denote no significant difference with $P > 0.05$, ** $P < 0.01$ (two-tailed Student's *t*-test). Source data are provided as a Source Data file.

Supplementary Figure 3

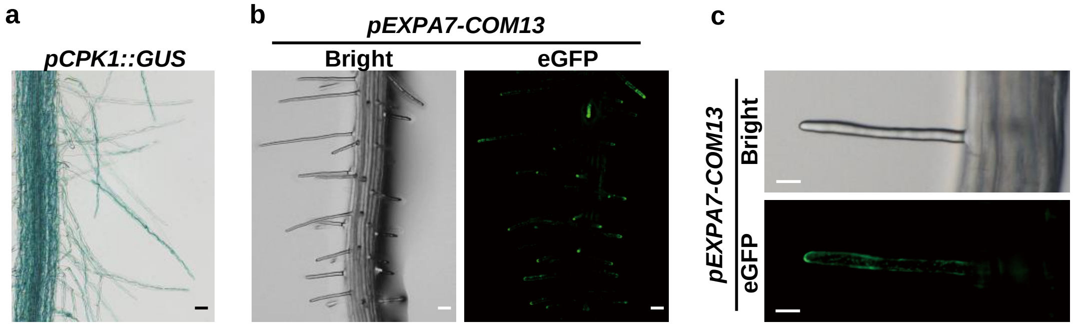

Supplementary Fig. 3 │ *CPK1* is highly expressed in RHs, and CPK1 is localized in the periphery of the RH apex. A set of bright-field or fluorescent optical section images were captured under a stereo microscope at room temperature (25 ± 1 °C), and were then merged into a single bright-field or fluorescent 2-D photo. This technique allows RHs growing in diverse directions around the primary roots to be clearly visualized. **a,** A typical merged 2-D photo of GUS staining results shows the expression of CPK1 in Arabidopsis RHs. **b**, Typical merged 2-D bright field (Left) and eGFP fluorescent (Right) photos of the root show that CPK1-eGFP localizes mainly at the RH apex. **c**, Typical bright field (Upper) and eGFP fluorescent (Lower) photos show the subcellular localization of CPK1-eGFP in the periphery of the RH apex. Scale bar, 0.1 mm in (**a-b**) and 50 μm in (**c**). 15 seedlings were analyzed in (**a**) and (**b**)**,** and 10 RHs were analyzed in (**c**).

Supplementary Figure 4

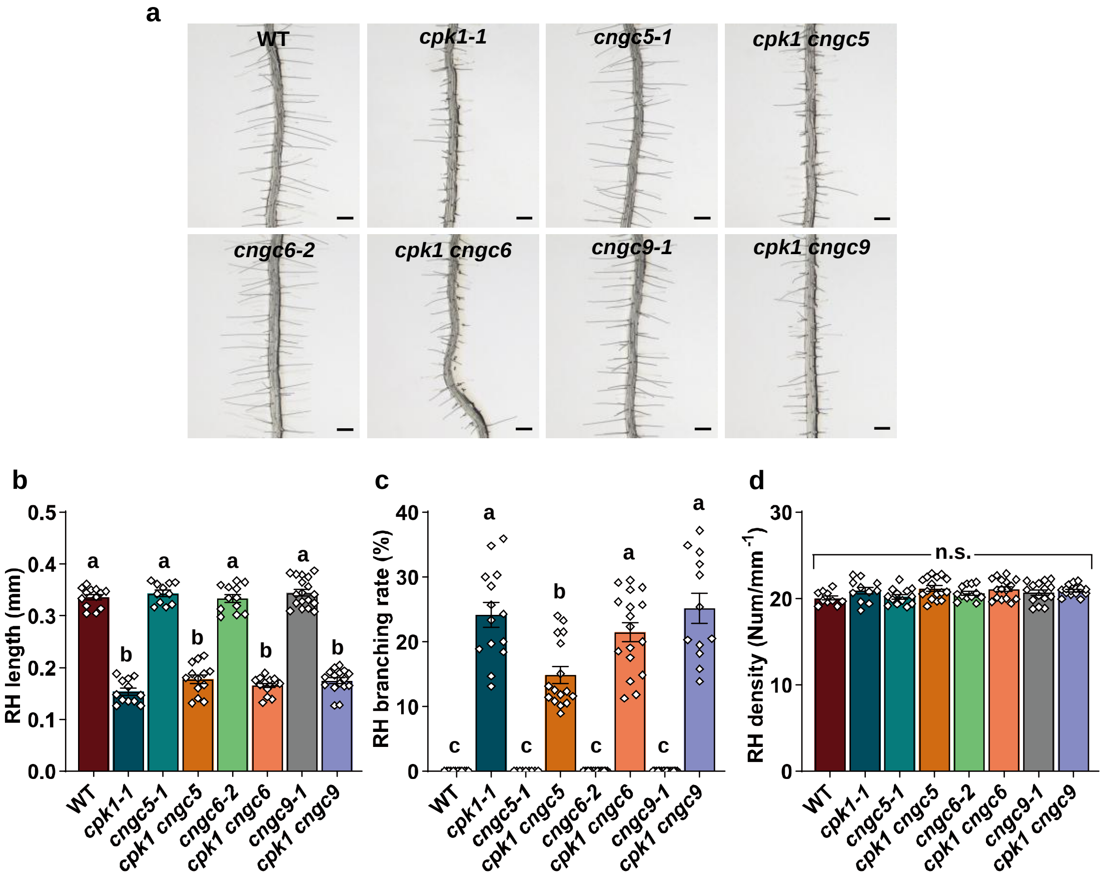

Supplementary Fig. 4 │ CPK1 functions upstream of CNGC5/6/9 to promote RH growth in Arabidopsis. For each seedling a set of images from optical sectioning of the RHZ of 4-5 day-old seedlings were captured under a stereo microscope at room temperature (25 ± 1 °C), and were merged into a 2-D picture. The merged 2-D pictures were used for the analysis of RH phenotypes. RH growth was not obviously impaired in the *cngc5-1*, *cngc6-2*, and *cngc9-1* single mutants, but the double mutants *cpk1-1 cngc5-1*, *cpk1-1 cngc6-2*, and *cpk1-1 cngc9-1* showed *cpk1-1*-like defects in RH growth. **a**, Typical merged 2-D pictures of the RHZ of seedlings. Scale bar, 0.2 mm. **b-d**, Statistical assay data of RH length (**b**), RH branching rates (**c**), and RH density (**d**). *cpk1 cngc5*, *cpk1 cngc6*, and *cpk1 cngc9* represent the double mutants *cpk1-1 cngc5-1*, *cpk1-1 cngc6-2*, and *cpk1-1 cngc9-1*, respectively. **b-d** The numbers of biologically independent roots with approximate 50 RHs per root examined are 13, 12, 11, 13, 12,

14, 18, and 16 for RH length (**b**), 12, 14, 9, 16, 15, 17, 15, and 12 for RH branching (**c**), and 10, 11, 13, 16, 12, 16, 15, and 15 for RH density (**d**), for the Arabidopsis lines as shown from left to right in each panel. Samples with different letters are significantly different with $P < 0.05$ (one-way ANOVA), and data are presented as means ± SEM, in (**b-d**). Source data are provided as a Source Data file.

Supplementary Figure 5

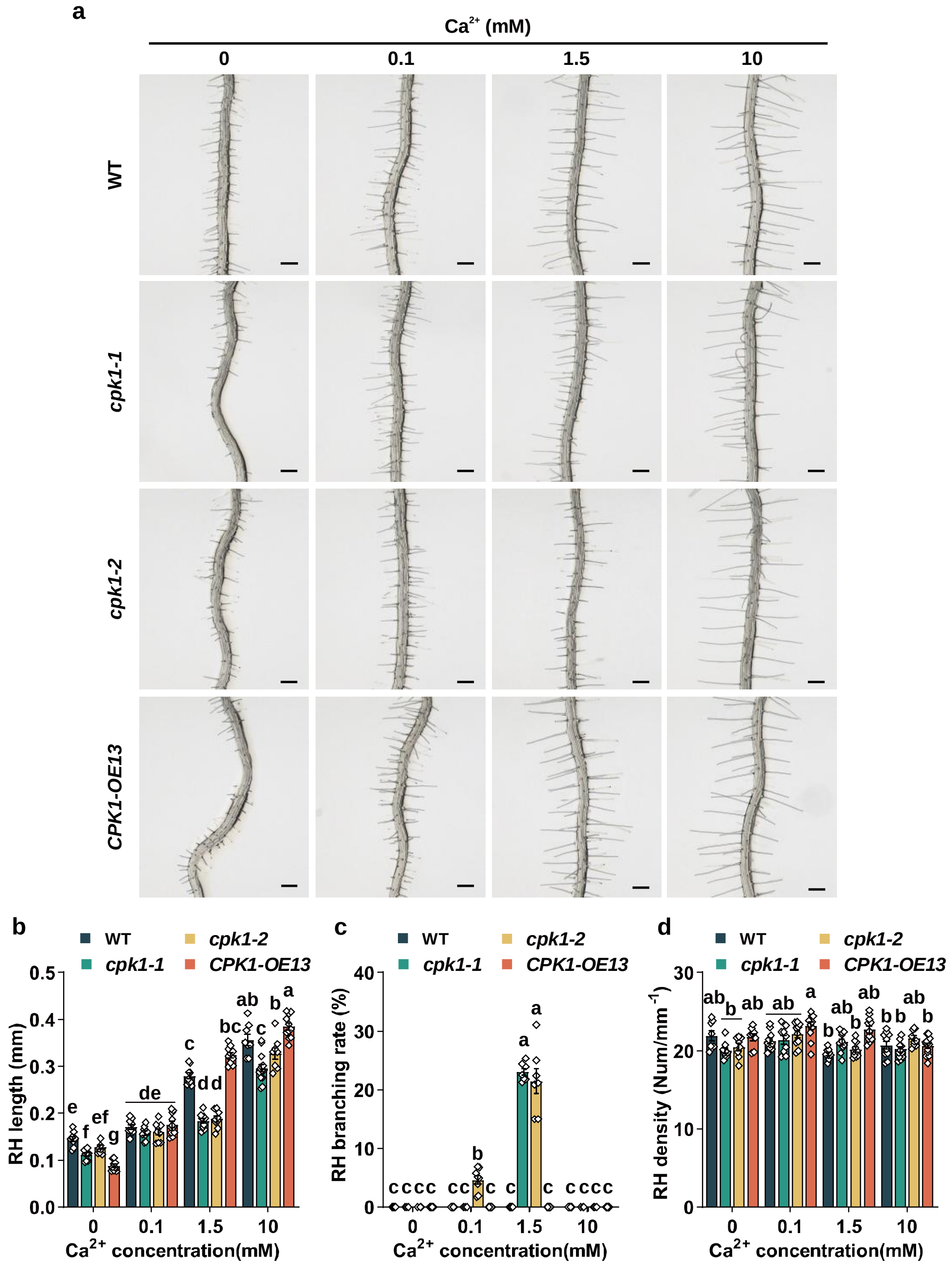

Supplementary Fig. 5 │ RH phenotypes in *cpk1-1* and *cpk1-2* can be rescued partially by high external Ca^{2+}. A series of optical section images of the RHZ of 4-5 day-old seedlings were captured under a stereo microscope at room temperature (25 ±

1 °C), and were merged into 2-D pictures that were used to analyze RH phenotypes. The RH phenotypes of *cpk1-1* and *cpk1-2* were partially rescued by high (10 mM) external Ca^{2+}. **a**, Typical merged 2-D pictures of seedling RHZ. **b-d**, Statistical analysis data of RH length (**b**), RH branching rates (**c**), and RH density (**d**). Scale bar, 0.2 mm in (**a**). Samples with different letters are significantly different with $P < 0.05$ (one-way ANOVA), and data are presented as means ± SEM, in (**b-d**). **b-d** Numbers of biologically independent roots with approximate 50 RHs per root examined are 9, 9, 9, 8, 9, 8, 10, 14, 9, 9, 9, 8, 9, 11, 10, and 11 for RH length (**b**), 9, 10, 9, 10, 10, 10, 7, 13, 8, 8, 7, 10, 8, 12, 12, and 13 for RH branching (**c**), 8, 10, 9, 10, 9, 9, 10, 12, 8, 10, 10, 9, 8, 11, 11, and 13 for RH density (**d**), for the Arabidopsis lines as shown from left to right in each panel. Source data are provided as a Source Data file.

Supplementary Figure 6

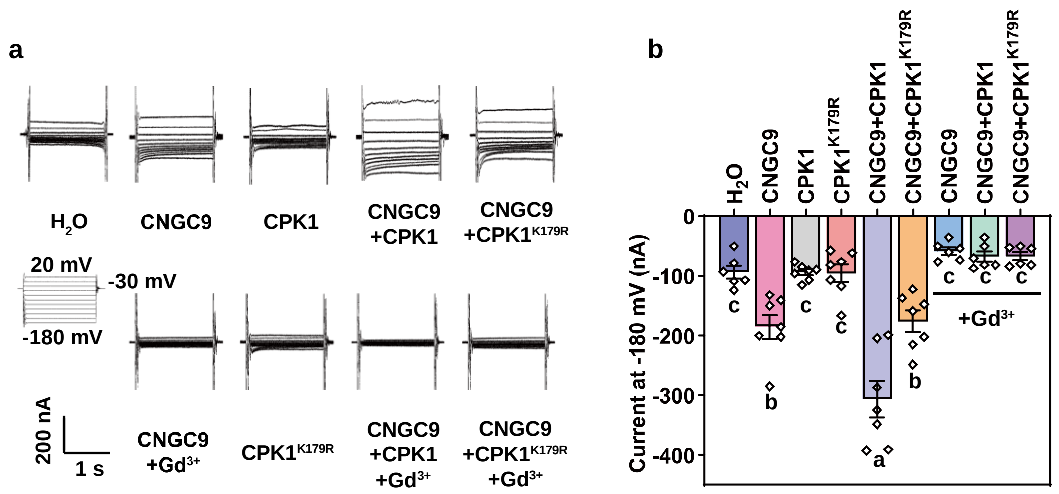

Supplementary Fig. 6 │ CPK1 activates CNGC9 in *Xenopus laevis* oocytes. cRNAs were prepared *in vitro*, and were microinjected into the oocytes separately or in combination as indicated, for transient gene expression. The oocytes were incubated at 16 ± 0.5 °C for 2-3 days after micro-injection, and then voltage clamping analysis was performed in the oocytes. A modest activation of CNGC9 was observed in the oocytes expressing CNGC9 alone, and an obviously activation of CNGC9 by CPK1 was observed in oocytes coexpressing CNGC9 and CPK1, relative to the tiny background conductance in the control oocytes injected with H_2O and the oocytes expressing either CPK1 or CPK1^{K179R} alone. Modest whole-oocyte currents were observed in oocytes coexpressing CNGC9 and loss-of-function CPK1^{K179R}, and those currents were similar to those of oocytes expressing CNGC9 alone, suggesting that CNGC9 was not activated by CPK1^{K179R}. The inward whole-oocyte currents mediated by CNGC9 were strongly inhibited by the Ca^{2+} channel blocker Gd^{3+} (100 µM). **a-b**, Typical whole-oocyte recordings (**a**) and statistical analysis of whole-cell currents at -180 mV (**b**). The numbers of oocytes tested are 6 for mock control, 7 for CNGC9, 7 for CPK1, 7 for CPK1^{K179R}, 7 for CNGC9+CPK1, 7 for CNGC9+CPK1^{K179R}, 6 for CNGC9+Gd^{3+}, 6 for CNGC9+CPK1+Gd^{3+}, and 6 for CNGC9+CPK1^{K179R}+Gd^{3+}. Data are presented as means ± SEM in (**b**). The samples with different letters denote significant difference with $P < 0.05$ (one-way ANOVA) in (**b**). Source data are provided as a Source Data file.

Supplementary Figure 7

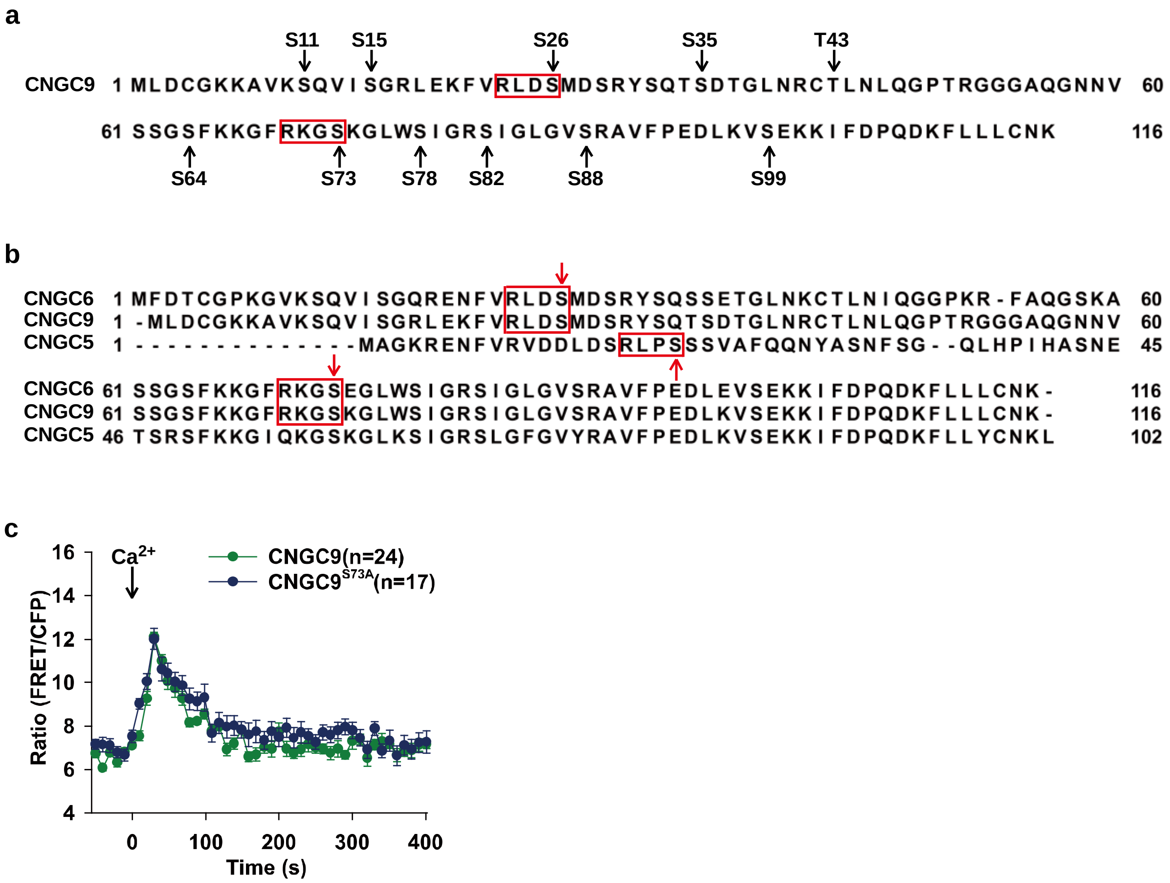

Supplementary Fig. 7 │ The identification of CPK1-target sites at the N termini of CNGC5/6/9. a, Multiple phosphorylation sites were identified in the CNGC9-N using LC-MS/MS analysis, including S26 and S73. **b**, Alignment of the CNGC5/6/9 N-termini revealed the serine sites in CNGC5 and CNGC6 correspond to the S26 and S73 in CNGC9. Arrows denote the candidate sites for CPK1 phosphorylation. **c**, Cytosolic Ca^{2+} imaging analysis in HEK293T cells show that the Ser73Ala point mutation in CNGC9 did not substantially alter the Ca^{2+} channel activity of CNGC9 in HEK293T cells. The letters n denote the numbers of HEK293T cells tested, and data are presented as means ± SEM in (**c**). Source data are provided as a Source Data file.

Supplementary Figure 8

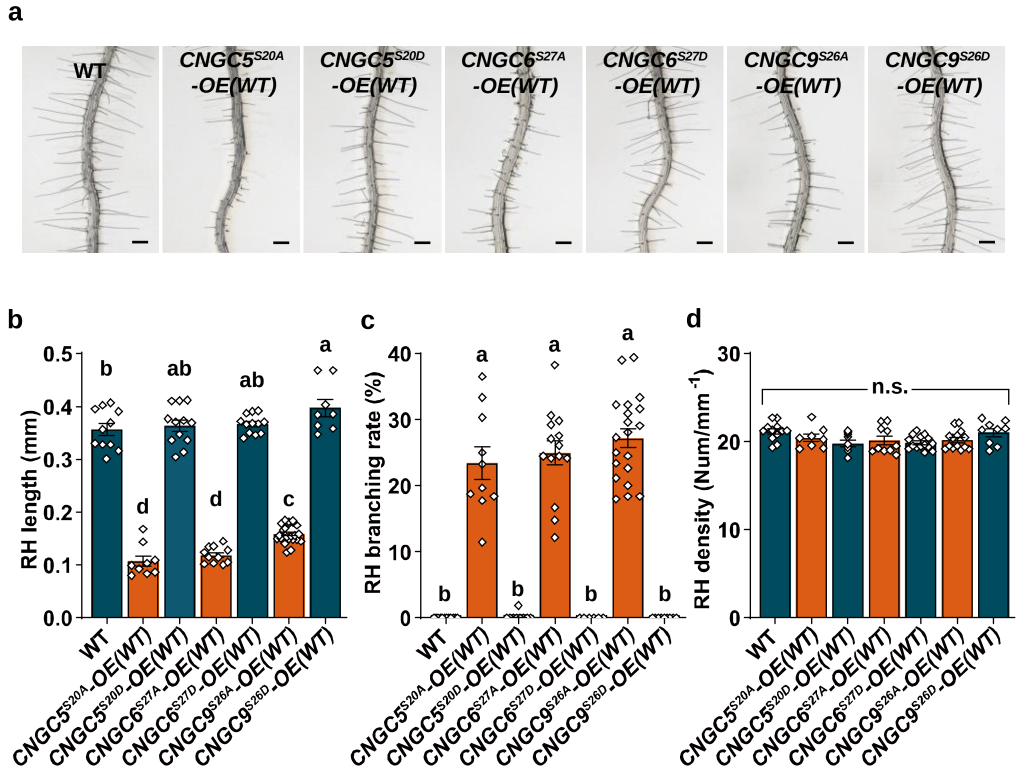

Supplementary Fig. 8 │ RH growth is strongly impaired by the overexpression of *iCNGCs*, but is not obviously affected by the overexpression of *aCNGCs*, under the *UBQ10* promoter in the wild type background. A set of images from optical sections of the RHZ with were captured under a stereo microscope at room temperature (25 ± 1 °C), and were merged into a 2-D picture. The merged 2-D pictures were used for the analysis of RH phenotypes. **a**, Typical merged 2-D pictures show the RHs of wild type and the transgenic Arabidopsis lines. **b-d**, The statistical analyses of RH length (**b**), RH branching rates (**c**), and RH density (**d**). Scale bar, 0.2 mm in (**a**). **b-d** The numbers of biologically independent roots with approximate 50 RHs per root examined are 11, 9, 12, 11, 12, 23, and 8 for RH length (**b**), 12, 10, 10, 14, 10, 20, and 10 for RH branching (**c**), and 12, 8, 11, 10, 15, 14, and 9 for RH density (**d**), for the Arabidopsis lines from left to right as shown in each panel. Samples with different letters are significantly different with $P < 0.05$ (one-way ANOVA), and data are presented as means ± SEM, in (**b-d)**. Source data are provided as a Source Data file.

Supplementary Figure 9

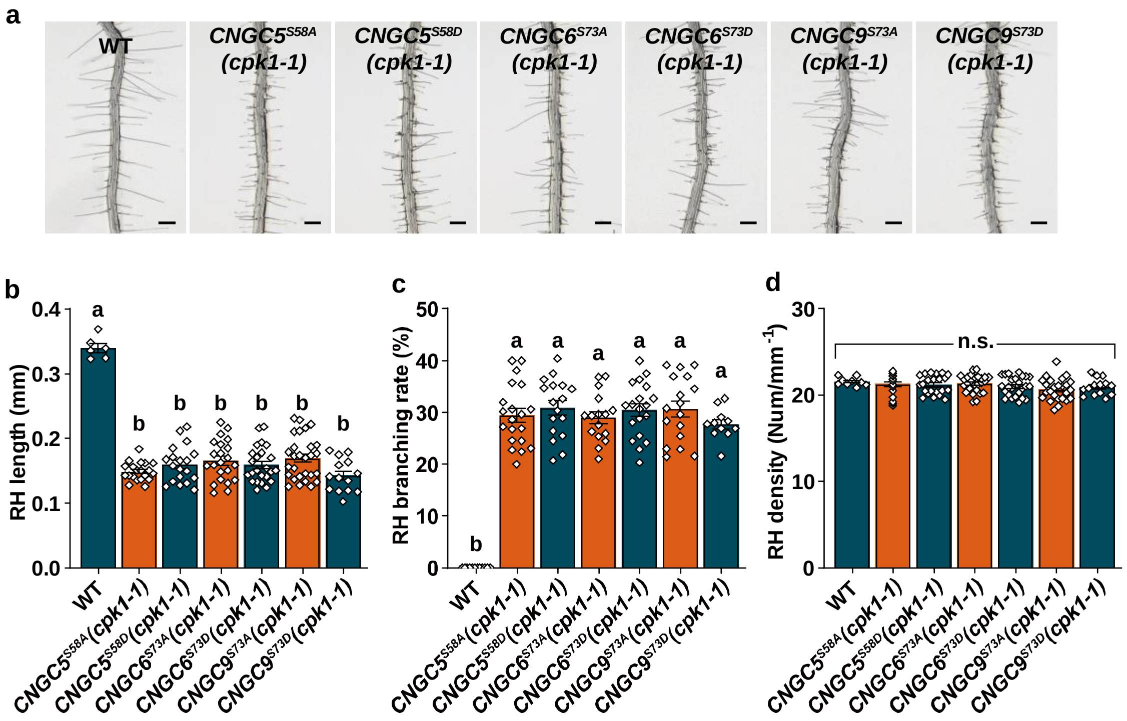

Supplementary Fig. 9 │ The expression of CNGC5/6/9 with point-mutations at secondary CPK1-target sites does not rescue the RH phenotypes of *cpk1-1*. A set of optical section images of the RHZ were captured under a stereo microscope at room temperature (25 ± 1 °C) for each Petri dish, and were merged into a 2-D picture. The merged 2-D pictures were used for RH phenotype analysis. **a**, Typical merged 2-D pictures of Arabidopsis seedlings show *cpk1-1*-like short RHs. **b-d**, Statistical analysis of RH length (**b**), RH branching rates (**c**), and RH density (**d**). Scale bar, 0.2 mm in (**a**). **b-d** Numbers of biologically independent roots with approximate 50 RHs per root examined are 6, 20, 19, 22, 24, 28, and 14 for RH length (**b**), 12, 20, 17, 16, 19, 17, and 11 for RH branching (**c**), and 9, 24, 22, 26, 25, 25, and 14 for RH density (**d**), for the Arabidopsis lines from left to right as shown in each panel. Samples with different letters are significantly different with $P < 0.05$ (one-way ANOVA), and data are presented as means ± SEM, in (**b-d)**. Source data are provided as a Source Data file.

Supplementary Figure 10

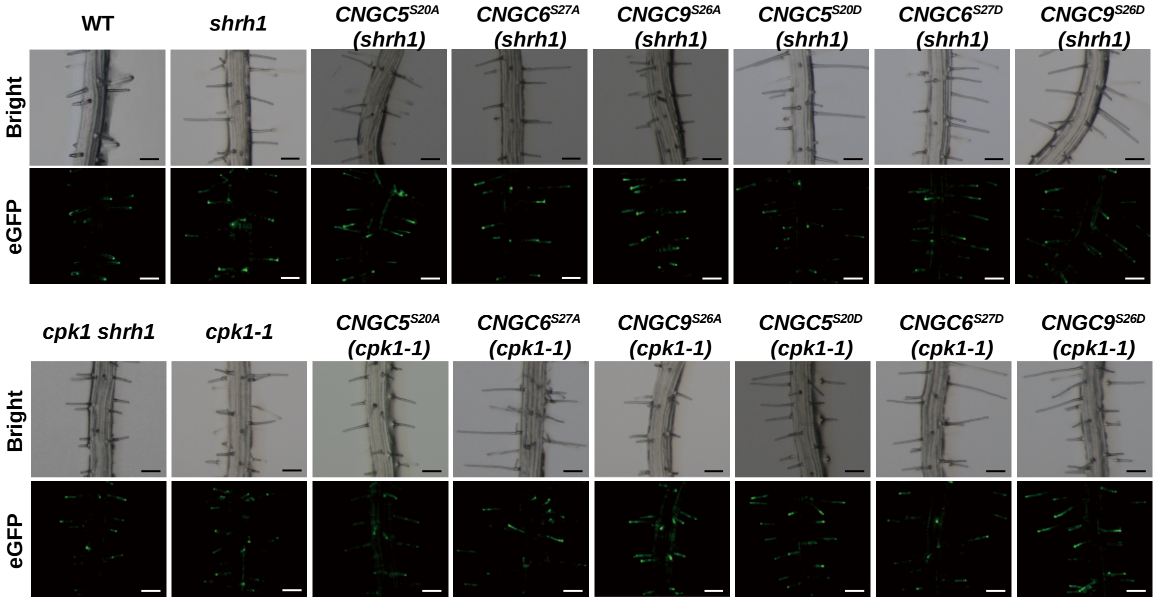

Supplementary Fig. 10 │The Ca^{2+} indicator YC3.6 is expressed at a similar level in the transgenic Arabidopsis lines used in this study. A Set of bright-field optical sectioning images and a set of fluorescent optical sectioning images of the RHZ were captured in turn under a stereo microscope at room temperature (25 ± 1 °C) for each seedling, and were further merged into a bright-field picture and a fluorescent 2-D picture, respectively. The typical merged 2-D fluorescent photos show the similar YFP fluorescent signal at the apex of RHs of the Arabidopsis lines, suggesting that the Ca^{2+} indicator YC3.6 is expressed at a similar level in those lines. Scale bar, 0.1 mm. 30 roots were analyzed for each Arabidopsis line.

Supplementary Table 1. List of Arabidopsis kinase mutants.

***Arabidopsis* mutant**	**Gene ID**	**Salk number**
cpk1-1	AT5G04870	SALK_080155
cpk1-2	AT5G04870	SALK_010530C
cpk2	AT3G10660	SALK_059237
cpk3	AT4G23650	SALK_107620
cpk4	AT4G09570	SALK_081860C
cpk6	AT2G17290	SALK_093308
cpk7	AT5G12480	SALK_127223C
cpk8	AT5G19450	SALK_036581C
cpk9	AT3G20410	SALK_019692C
cpk10	AT1G18890	SALK_032021
cpk11	AT1G35670	SALK_023086
cpk12	AT5G23580	SALK_090011C
cpk14	AT2G41860	SALK_100193C
cpk15	AT4G21940	CS841768
cpk17	AT5G12180	SALK_140527C
cpk18	AT4G36070	SALK_061352C
cpk20	AT2G38910	SALK_044320C
cpk22	AT4G04710	SALK_125850C
cpk24	AT2G31500	SALK_015986C
cpk27	AT4G04700	SALK_141648C
cpk28	AT5G66210	SALK_112540C
cpk29	AT1G76040	SALK_114657
cpk30	AT1G74740	SALK_108447C
cpk32	AT3G57530	SALK_012340C
cpk33	AT1G50700	SALK_059467C
cpk34	AT5G19360	SALK_040605
snrk2.2	AT3G50500	GABI-Kat 807G04
snrk2.3	AT5G66880	SALK_107315
snrk2.2/2.3	-	-
snrk2.2/2.3/2.6	-	-
snrk2.4	AT1G10940	SALK_075889
snrk2.6/ost1	AT4G33950	SALK_067550
snrk2.7	AT4G40010	SALK_042338
snrk2.8	AT1G78290	SALK_069354C
mpk5	AT4G11330	SALK_129907
mapkkk17	AT2G32510	SALK_080309C
cbl1	AT4G17615	SALK_110426C
cbl9	AT5G47100	SALK_142774
cbl1/cbl9-1	-	-
cipk23-1	AT1G30270	SALK_036154
