# Supplementary material for: CPK1 activates CNGCs through phosphorylation for Ca2+ signaling to promote root hair growth in Arabidopsis
Source: Nat Commun. 2025 Jan 15;16:676. doi: 10.1038/s41467-025-56008-4 (PMC11733299; doi:10.1038/s41467-025-56008-4)
Supplement: Supplementary file 3 — Description of Additional Supplementary Files [file 41467_2025_56008_MOESM3_ESM.pdf]

Description of Additional Supplementary Files

Supplementary Data 1: Primers for RT-PCR, qRT-PCR, and vector construction.
