# Supplementary material for: CPK1 activates CNGCs through phosphorylation for Ca2+ signaling to promote root hair growth in Arabidopsis
Source: Nat Commun. 2025 Jan 15;16:676. doi: 10.1038/s41467-025-56008-4 (PMC11733299; doi:10.1038/s41467-025-56008-4)
Supplement: Supplementary file 5 — Reporting Summary [file 41467_2025_56008_MOESM5_ESM.pdf]

Corresponding author(s): Yong-Fei Wang

Last updated by author(s): Dec 17, 2024

Reporting Summary

Nature Portfolio wishes to improve the reproducibility of the work that we publish. This form provides structure for consistency and transparency in reporting. For further information on Nature Portfolio policies, see our Editorial Policies and the Editorial Policy Checklist.

Statistics

For all statistical analyses, confirm that the following items are present in the figure legend, table legend, main text, or Methods section.

n/a	Confirmed	
☐	☒	The exact sample size (*n*) for each experimental group/condition, given as a discrete number and unit of measurement
☐	☒	A statement on whether measurements were taken from distinct samples or whether the same sample was measured repeatedly
☐	☒	The statistical test(s) used AND whether they are one- or two-sided<br>*Only common tests should be described solely by name; describe more complex techniques in the Methods section.*
☐	☒	A description of all covariates tested
☐	☒	A description of any assumptions or corrections, such as tests of normality and adjustment for multiple comparisons
☐	☒	A full description of the statistical parameters including central tendency (e.g. means) or other basic estimates (e.g. regression coefficient) AND variation (e.g. standard deviation) or associated estimates of uncertainty (e.g. confidence intervals)
☐	☒	For null hypothesis testing, the test statistic (e.g. *F*, *t*, *r*) with confidence intervals, effect sizes, degrees of freedom and *P* value noted<br>*Give P values as exact values whenever suitable.*
☒	☐	For Bayesian analysis, information on the choice of priors and Markov chain Monte Carlo settings
☒	☐	For hierarchical and complex designs, identification of the appropriate level for tests and full reporting of outcomes
☒	☐	Estimates of effect sizes (e.g. Cohen's *d*, Pearson's *r*), indicating how they were calculated

Our web collection on statistics for biologists contains articles on many of the points above.

Software and code

Policy information about availability of computer code

Data collection	pClamp 10.02 (Axon Instrument, USA); MetaFluor 7.8.0.0 (Molecular Devices, USA); Leica Application Suite X (Leica, Germany).
Data analysis	SigmaPlot 11.0 (Systat Tech., USA); pClamp 10.02 (Axon Instrument, USA); MetaFluor 7.8.0.0 (Molecular Devices, USA); GraphPad Prism 9.0 (GraphPad Sotware, USA).

For manuscripts utilizing custom algorithms or software that are central to the research but not yet described in published literature, software must be made available to editors and reviewers. We strongly encourage code deposition in a community repository (e.g. GitHub). See the Nature Portfolio guidelines for submitting code & software for further information.

Data

Policy information about availability of data

All manuscripts must include a data availability statement. This statement should provide the following information, where applicable:

- Accession codes, unique identifiers, or web links for publicly available datasets
- A description of any restrictions on data availability
- For clinical datasets or third party data, please ensure that the statement adheres to our policy

Numerical source data giving rise to graphical representation for each data point for main and extended data figures are provided as Source Data. PCR primers and vectors are provided in Materials and Methods of the main manuscript and Supplementary Data Set 2. All other data and materials supporting the findings of this

study are available in the main text or the supplementary materials. Source data are provided with this paper.

Research involving human participants, their data, or biological material

Policy information about studies with human participants or human data. See also policy information about sex, gender (identity/presentation), and sexual orientation and race, ethnicity and racism.

Reporting on sex and gender	n/a
Reporting on race, ethnicity, or other socially relevant groupings	n/a
Population characteristics	n/a
Recruitment	n/a
Ethics oversight	n/a

Note that full information on the approval of the study protocol must also be provided in the manuscript.

Field-specific reporting

Please select the one below that is the best fit for your research. If you are not sure, read the appropriate sections before making your selection.

☒ Life sciences ☐ Behavioural & social sciences ☐ Ecological, evolutionary & environmental sciences

For a reference copy of the document with all sections, see nature.com/documents/nr-reporting-summary-flat.pdf

Life sciences study design

All studies must disclose on these points even when the disclosure is negative.

Sample size	No statistical method was used to predetermine sample size. Sample size was chosen as large as possible and in accordance with previous established protocols in the field and references cited in the Methods section.
Data exclusions	No data were excluded from the analysis.
Replication	The recordings and parameters were reproducible across multiple days and batches, which was dependent on the experiments. Ca2+ imaging and patch clamping experiments and the analysis of root hair-related phenotypes were repeated from weeks to years.
Randomization	All samples collected randomly were used for data analysis.
Blinding	Blinding was not relevant to this study. Experiments were repeated as more as possible throught years.

Reporting for specific materials, systems and methods

We require information from authors about some types of materials, experimental systems and methods used in many studies. Here, indicate whether each material, system or method listed is relevant to your study. If you are not sure if a list item applies to your research, read the appropriate section before selecting a response.

Materials & experimental systems

n/a	Involved in the study	
☐	☒	Antibodies
☐	☒	Eukaryotic cell lines
☒	☐	Palaeontology and archaeology
☐	☒	Animals and other organisms
☒	☐	Clinical data
☒	☐	Dual use research of concern
☐	☒	Plants

Methods

n/a	Involved in the study	
☒	☐	ChIP-seq
☒	☐	Flow cytometry
☒	☐	MRI-based neuroimaging

Antibodies

Antibodies used	Antibodies against the tags Myc (Catalog # Ab62928, Ambcam) and Flag (Catalog # M20008, Abmart) were purchased from vendors.
Validation	No validation was done after the purchase of the antibodies from the vendors.

Eukaryotic cell lines

Policy information about cell lines and Sex and Gender in Research

Cell line source(s)	HEK293T cells were purchased from vendors.
Authentication	No further authentication of the cell line was done after purchasing.
Mycoplasma contamination	No further test for mycoplasma contamination of the cell line was done after purchasing.
Commonly misidentified lines (See ICLAC register)	The HEK293T cell line used in this study is not amongst the misidentified cell lines.

Animals and other research organisms

Policy information about studies involving animals; ARRIVE guidelines recommended for reporting animal research, and Sex and Gender in Research

Laboratory animals	Frogs (Xenopus laevis) were from Shen's lab at Hangzhou Normal University (Hangzhou City, China).
Wild animals	n/a
Reporting on sex	n/a
Field-collected samples	n/a
Ethics oversight	n/a

Note that full information on the approval of the study protocol must also be provided in the manuscript.

Dual use research of concern

Policy information about dual use research of concern

Hazards

Could the accidental, deliberate or reckless misuse of agents or technologies generated in the work, or the application of information presented in the manuscript, pose a threat to:

No	Yes	
☒	☐	Public health
☒	☐	National security
☒	☐	Crops and/or livestock
☒	☐	Ecosystems
☒	☐	Any other significant area

Experiments of concern

Does the work involve any of these experiments of concern:

No	Yes	
☒	☐	Demonstrate how to render a vaccine ineffective
☒	☐	Confer resistance to therapeutically useful antibiotics or antiviral agents
☒	☐	Enhance the virulence of a pathogen or render a nonpathogen virulent
☒	☐	Increase transmissibility of a pathogen
☒	☐	Alter the host range of a pathogen
☒	☐	Enable evasion of diagnostic/detection modalities
☒	☐	Enable the weaponization of a biological agent or toxin
☒	☐	Any other potentially harmful combination of experiments and agents

Plants

Seed stocks	Arabidopsis seeds of wild type and single T-DNA insertional mutants were originally purchased from ABRC, and the other lines were generated by the researchers in the laboratory.
Novel plant genotypes	No novel plant genotype was generated in this study.
Authentication	No authentication was done after the seeds were purchased from ABRC.
